# Swiftly Computing Center Strings

**DOI:** 10.1186/1471-2105-12-106

**Published:** 2011-04-19

**Authors:** Franziska Hufsky, Léon Kuchenbecker, Katharina Jahn, Jens Stoye, Sebastian Böcker

**Affiliations:** 1 Lehrstuhl für Bioinformatik, Friedrich-Schiller-Universität Jena, Ernst-Abbe-Platz 2, Jena, Germany; 2 Max Planck Institute for Chemical Ecology, Beutenberg Campus, Jena, Germany; 3 AG Genominformatik, Technische Fakultät, Universität Bielefeld, Bielefeld, Germany

## Abstract

**Background:**

The center string (or closest string) problem is a classic computer science problem with important applications in computational biology. Given *k *input strings and a distance threshold *d*, we search for a string within Hamming distance at most *d *to each input string. This problem is NP complete.

**Results:**

In this paper, we focus on exact methods for the problem that are also swift in application. We first introduce data reduction techniques that allow us to infer that certain instances have no solution, or that a center string must satisfy certain conditions. We describe how to use this information to speed up two previously published search tree algorithms. Then, we describe a novel iterative search strategy that is effecient in practice, where some of our reduction techniques can also be applied. Finally, we present results of an evaluation study for two different data sets from a biological application.

**Conclusions:**

We find that the running time for computing the optimal center string is dominated by the subroutine calls for *d *= *d*_opt _-1 and *d *= *d*_opt_. Our data reduction is very effective for both, either rejecting unsolvable instances or solving trivial positions. We find that this speeds up computations considerably.

## Background

The CENTER STRING problem (also known as CLOSEST STRING problem) is defined as follows: given *k *strings of length *L *over an alphabet Σ and a distance threshold *d*, find a string of length *L *that has Hamming distance at most *d *to each of the given strings.

The CENTER STRING problem has been studied extensively in theoretical computer science and, particularly, in computational biology [1,2], and has various applications such as degenerate PCR primer design [3] or motif finding [1,4]. We are particularly interested in its application as part of finding approximate gene clusters. The increasing speed of genome sequencing and the resulting increase in the number of available data sets offers the possibility of comparing the gene order of whole genomes. During the course of evolution, speciation results in the divergence of genomes that initially have the same gene order and content. Conserved gene order is evidence of a particular biological signal [5]. Approximate gene cluster models account for reordering inside the gene cluster, as well as additional and missing genes in the genomes compared [6,7]. The *center gene cluster model *limits the distance between the gene cluster and each of the approximate occurrences. For given approximate occurrences, finding the center gene cluster is equivalent to finding a center string for binary input strings.

### Previous work

The CENTER STRING problem is NP complete [1,8], hence no polynomial time algorithm can exist unless P = NP. Different approaches have been studied for the problem. Ma and Sun [9] presented a polynomial time approximation scheme with time complexity  for an approximation ratio of 1 + *ε *for any *ε *> 0. In addition, heuristics and parallel implementations with good practical running times have been developed [10,11]. The drawback of these approaches is that they cannot guarantee that an exact solution will be found.

In parameterized algorithmics, we use a parameter to describe the complexity of a problem instance. We restrict the super-polynomial running time of an algorithm using this parameter while at the same time still guaranteeing that optimal solutions are found. Formally, a problem with input size *n *and parameter *k *is *fixed-parameter tractable *if it can be solved in *O*(*f*(*k*) · *p*(*n*)) time, where *f *is an arbitrary function and *p *is a polynomial. Parameters that have been studied in the literature for the CENTER STRING problem are the distance threshold *d *and the number of input strings *k*. For the latter parameter, Gramm *et al. *[12] showed that the problem is fixed-parameter tractable using an Integer Linear Program. Evaluations indicate that this approach is of theoretical interest only and impractical for *k *≥ 5. Regarding the distance threshold *d*, in the same paper an algorithm was given with running time *O*(*kL *+ *kd*^*d*+1^). Later, Ma and Sun [9] presented an algorithm with running time *O*(*kL *+ *kd *· 16^*d*^(|Σ| - 1)*^d^*). Recently, Wang and Zhu [2] further improved running times to *O*(*kL *+ *kd *· 9.53*^d^*(|Σ| - 1)*^d^*), and Chen *et al. *[13] to *O*(*kL *+ *kd*^2^6.74*^d^*) for binary strings. These algorithms are based on the search tree paradigm. Note that for binary strings, the term (|Σ| - 1)*^d ^*vanishes.

Besides these fixed-parameter approaches, Meneses *et al. *[14] proposed a heuristic to compute upper and lower bounds using a branch-and-bound algorithm and, very recently, Kelsey and Kotthoff [15] investigated a constraint programming approach.

All of the above results, as well as our results presented below, deal with the CENTER STRING problem under the Hamming distance. Nicolas and Rivals [16] showed that the CENTER STRING problem under the Levenshtein distance is NP-hard and W[1]-hard regarding the number of input strings, even for binary strings. On this account, no FPT algorithm with parameter *k *can exist unless FPT = W[1]. Furthermore, the authors generalized these results to any weighted edit distance satisfying a certain natural condition, namely, a slightly tightened triangle inequality (see Property 1 in [16] for details). Note, that CENTER STRING is polynomial if the number of input strings and the weighted edit distance are fixed [16].

### Our contribution

In this paper, we focus on exact methods that are also swift in application. We have developed an advanced preprocessing to filter out unsolvable instances quickly. Additionally, we compute rules that can be used within search tree algorithms to bound the search space, excluding unsolvable instances. We show how to integrate this information into the algorithms from [9,12]. We then present a new iterative search strategy called *MismatchCount*, which, despite its bad worst-case running time, works well in practice. We implemented all three algorithms to evaluate their performance in combination with our preprocessing. We present results of our experimental evaluation, showing that preprocessing and the novel algorithm improve running times by several orders of magnitude. We find that, in particular, the cases *d *= *d*_opt _- 1 and *d *= *d*_opt _are notoriously difficult for all approaches, where *d*_opt _is the smallest distance value for which a solution exists.

A preliminary version of this paper has been published in Proc. of Workshop on Algorithms in Bioinformatics, WABI 2010, Volume 6293 of Lect. Notes Comput. Sc., Springer 2010:325-336.

## Methods

### Preliminaries

For a string *s *over a finite alphabet Σ, let *s*[*i*] be the *i*th character of *s *and *s*[*i*, *j*] the substring of *s *starting at position *i *and ending at position *j*. The length of *s *is denoted by |*s*|.

The *Hamming distance d*_H_(*s*, *t*) of two strings *s *and *t *of the same length *L *is the number of positions *p *with *s*[*p*] ≠ *t*[*p*]. Let *R *= {*p*_1_,..., *p*_*m*_} ⊆ {1,..., *L*} be a set of positions such that *p*_*i *_<*p*_*i*+1 _for all 1 ≤ *i *<*m*. Then *s*|_*R *_:= *s*[*p*_1_] ... *s*[*p*_*m*_] denotes the subsequence of *s *restricted to the positions in *R*. We define the Hamming distance of two strings *s *and *t *restricted to *R *as . For two strings *s *and *t*, let *D*_*s*, *t *_:= {*p *: *s*[*p*] ≠ *t*[*p*]} ⊆ {1,..., *L*} be the set of positions where *s *and *t *differ, and let *E*_*s, t *_:= {*p *: *s*[*p*] = *t*[*p*]} = {1,..., *L*}\*D*_*s*, *t *_be the set of positions where *s *and *t *are identical. Note that . For *k *input strings *s*_1_,..., *s*_*k*_, we write  and . As noted in the introduction, we will often limit ourselves to a binary alphabet Σ = {0, 1}, here, we define .

The CENTER STRING problem is defined as follows: for strings *s*_1_,..., *s*_*k *_of length *L *over an alphabet Σ, and a distance threshold *d*, find a string *ŝ *of length *L*, called *center string*, which has Hamming distances at most *d *to each of the given strings.

We note that permuting positions of all strings by *the same *permutation, results in an equivalent instance. Let *π *be a permutation over positions 1,..., *L*. For a string *s *= *s*(1) ... *s*(*L*) of length *L*, let *π*(*s*) := *s*(*π*(1)) *s*(*π*(2)) ... *s*(*π*(*L*)) be the permuted string. Let *s*_1_,..., *s_k _*be an instance of CENTER STRING problem, in which all strings have length *L*. Then, a given string *s *has Hamming distance at most *d *to all strings *s*_1_,..., *s_k_*, if and only if *π*(*s*) has Hamming distance at most *d *to all strings *π*(*s*_1_),..., *π*(*s_k_*). For *k *strings *s*_1_,..., *s_k _*and distance threshold *d*, we can construct a *naïve kernel *as follows [12]: a position *p *is called *clean *if all sequences coincide at this position, i.e. *s_i_*[*p*] = *s_j_*[*p*] for all 1 ≤ *i *<*j *≤ *k*. If a position is not clean, we call it *dirty*. One can easily see that there can be at most *kd *dirty positions if an instance with *k *strings allows for a center string with distance *d*. If a position is not dirty, then all strings share the same character at this position, and the center string will also share this character. We can thus remove all clean positions and obtain an instance of length *L *≤ *kd*. Now let us assume that *d *is given to us as a parameter. Again, we remove all clean positions from the instance. If the resulting strings have more than *kd *characters, the instance can be rejected. Similarly, we can reject an instance that contains a string pair with distance larger than 2*d*, since the Hamming distance is a metric and satisfies the triangle inequality. In our algorithms, we assume a distance threshold *d *to be given. In applications, we might not know the distance threshold *d *in advance but instead search for a center string minimizing *d*. We can do so by calling our algorithms repeatedly, increasing *d *= 0, 1, 2,... until a solution is found for *d *= *d*_opt_. Both in theory and in our experimental evaluation, we find that the running time of this iteration is governed by the last subroutine calls with *d *= *d*_opt _- 1 and *d *= *d*_opt_. To this end, we will put special focus on these two cases in our evaluations.

Our proposed data reduction often allows us to infer that no solution can exist for a particular distance threshold *d*. However, where we cannot rule out the existence of a center string by data reduction (what is obviously the case when *d *= *d*_opt_), we still have to decide whether a valid center string exists. All algorithms for doing so, such as those presented in [2,9,12] and the *MismatchCount *algorithm presented in this paper, scan through all 2*^L ^*possible binary strings and test whether any such string is a center string of the input. The algorithms differ in the order in which they process the 2*^L ^*strings and, in particular, how they constrain the search space to speed up computations.

### Data reduction

Our data reduction is based on the pairwise comparison of the input strings. Given an instance *s*_1_,..., *s_k _*and *d *of the CENTER STRING problem, we can divide all pairs of strings {*s_i_*, *s_j_*} into three groups: pairs with distance less than 2*d *- 1, greater than 2*d*, or equal to 2*d *or 2*d *- 1. If two strings *s_i_*, *s_j _*with Hamming distance *d*_H_(*s_i_*, *s_j_*) > 2*d *exist, then the instance has no solution. A center string *ŝ *can have at most distance *d *to each of *s_i _*and *s_j _*and, hence, *d*_H_(*s_i_*, *s_j_*) ≤ *d*_H_(*s_i_*, *ŝ*) + *d*_H_(*ŝ*, *s_j_*) ≤ 2*d*. Therefore,  must hold for the instance to have a solution.

#### Solving trivial positions

Some positions of the solution string can be trivially solved. This is based on the following observation:

**Lemma 1**. *Given strings s*_1_,..., *s_k _and a center string ŝ with distance d*. *For two strings s_i_*, *s_j _such that d*_H_(*s_i_*, *s_j_*) = 2*d or d*_H_(*s_i_*, *s_j_*) = 2*d *- 1, *we have*

*Proof*. A center string with distance at most *d *to all strings is located centrally between the two strings *s_i _*and *s_j _*with distance 2*d *and therefore has distance *d *to both of them. Thus, all positions fixed between *s_i _*and *s_j _*must also be fixed in *ŝ*. We can extend our reasoning to string pairs with distance 2*d *- 1. We need to change *d *positions in at least one of the strings and *E_i,j _*is the set of equal positions between *both *strings, hence we are still not allowed to change any position *p *∈ *E_i,j_*.

As a reduction rule, if we find two strings *s_i_*, *s_j _*with *d*_H_(*s_i_*, *s_j_*) ≥ 2*d *- 1, then we can set *ŝ*[*p*] := *s_i_*[*p*] for all *p *∈ *E_i, j _*and mark these positions as "permanent". Let  denote this set of permanent positions. We can generalize this rule to solve additional positions. Assume a specific *d_i _*:= *d *for each input string *s_i_*, which is increased by one for every solved position that does not match *s_i_*. If we find two strings *s_i_*, *s_j _*with *d*_H_(*s_i_*, *s_j_*) = *d_i _*+ *d_j_*, we can again set *ŝ*[*p*] := *s_i_*[*p*] for all *p *∈ *E_i, j _*and mark these positions as "permanent". In the case *d_i _*= *d_j _*the rule remains the same as that given above. We repeat this rule until no fitting string pair *s_i_*, *s_j _*can be found.

Applying this reduction rule, we may run into *conflicts *where we have to permanently set a certain position to '0' and '1' simultaneously. We infer that the instance has no solution for the current choice of *d*. If we do not have a conflict, then applying this data reduction results in a partially solved solution string *ŝ *with *ŝ*[*p*] = *c *∈ ∑ fixed for all , whereas all positions not in  still have to be decided.

#### Computation of position subsets

We focus next on pairs of strings *s_i_*, *s_j _*with *d*_H_(*s_i_*, *s_j_*) < 2*d *- 1. For a given center string *ŝ *we define

as the set of positions where *s_i _*and *s_j _*agree, but disagree with the center string *ŝ*. We extend the reasoning behind Lemma 1 as follows:

**Lemma 2**. *Given strings s*_1_,..., *s_k _and a center string ŝ with distance d*. *For two strings s_i_, s_j _such that d*_H_(*s_i_*, *s_j_*) <*2d *- *1, we have*

*Proof*. Set *D *:= *D*_*i*, *j*_. Regarding the distances between *ŝ*|_*D *_and *s*_*i*_|_*D *_as well as *s*_*j*_|_*D*_, we can state that *ŝ*|*_D _*has a distance of at least  to at least one of the strings *s*_*i*_|*_D _*or *s_j_*|*_D_*:

This is true since *d*_H _is a metric and the triangle inequality holds, *d*_H_(*s_i_*|*_D_*) ≤ *d*_H_(*s_i_*|*_D_*, *ŝ*|*_D_*) + *d*_H_(*s_j_*|*_D_*, *ŝ*|*_D_*). Since we need a distance of at least  to solve the positions from *D*, a distance of at most  remains to solve the positions from *E*_*i,j*_.

Lemma 2 implies that the maximum number of positions *p *∈ *E_i, j _*that we are allowed to choose in the center string with *ŝ*[*p*] ≠ *s_i_*[*p*] is bounded by . We can transform this observation into a reduction rule as follows: when, during search tree traversal or by other reduction rules, we have a partially solved solution string *ŝ *such that

for any pair *s_i_*, *s_j_*, then we can infer that *ŝ *cannot be extended to a solution for the current choice of *d*. For each pair *s_i_*, *s_j_*, we therefore set  and store all tuples (*E_i,j_*, *x_i,j_*) in an array .

Removing redundant information from  may lead to further trivially solved positions. This is done by removing, for all 1 ≤ *i *<*j *≤ *k*, all positions **from *E_i,j_*. Moreover, if *ŝ*[*p*] ≠ *s_i_*[*p*] then we decrease *x_i,j _*by one.

For *x*_*i,j *_= 0 we set all positions *p *from *E*_*i*,*j *_to "permanent" and include them in . Since  has changed, we continue our data reduction again until there is no tuple (*E*_*i,j*_, *x*_*i,j*_) with *x*_*i,j *_= 0 in . For *x*_*i,j *_< 0 we can easily infer that a conflict must exist and, as a result, the instance has no valid solution for this distance threshold *d*.

#### Cascading

To enlarge further the number of solved positions we consider all pairs of strings *s_i_*, *s_j _*with *x_i,j _*= 1 and use *cascading*. A valid center string *ŝ *has to agree with *s_i _*in at least |*E*_*i*,*j*_| - 1 positions from *E*_*i,j*_, hence for binary strings, at most one position *p *∈ *E*_*i,j *_can be set to  .

To this end, we test for all positions *p *∈ *E_i,j _*what we can infer from setting  This implies *x_i, j _*= 0, hence we add the remaining positions *q *∈ *E_i,j_*, *q *≠ *p*, to  and reduce the tuple set . If we run into a conflict during this reduction, we know that setting  cannot result in a valid solution. In this case, we infer *ŝ*[*p*] = *s_i_*[*p*] and permanently set position *p*.

Unfortunately, if there is no conflict, setting *ŝ*[*p*] = *s_i_*[*p*] is not mandatory. Nonetheless, we get a partially solved solution string *ŝ_p,v _*and a set of "potentially permanent" positions  depending on the position *p *and the value . We store this information in a set of rules .

We can use the set of rules  when solving the remaining instance, for example by means of a search tree algorithm. If, during the search tree traversal, we decide to set *ŝ*[*p*] = *v *for the solution string *ŝ*, then we can immediately start the above data reduction. For all positions , we set the solution string *ŝ*[*q*] = *ŝ_p,v_*[*q*]. For the remaining positions  the condition *ŝ*[*q*] = *ŝ_p,v_*[*q*] must be met, otherwise we run into a conflict and, thus, this branch of the search tree does not lead to a valid solution.

### Integration into search tree algorithms

We can use the information derived during preprocessing, stored in the sets , , , to speed up the algorithms of Ma and Sun [9], and Gramm *et al. *[12]. Unfortunately, the use of , ,  does not change the worst-case running times of both algorithms. But our preprocessing, as an algorithm engineering technique, allows us to speed up the algorithms in practice.

**The algorithm of Ma and Sun **tackles the more general NEIGHBOR STRING problem. Given *s*_1_, *s*_2_,..., *s_k _*of length *L *and non-negative integers *d*_1_, *d*_2_,..., *d_k_*, find a string *ŝ *of length *L *such that *d*(*ŝ*, *s_i_*) ≤ *d_i _*for every 1 ≤ *i *≤ *k*. The algorithm starts by testing whether *s*_1 _is already a valid solution. If not, there has to be at least one  with . For these two strings *s*_1 _and . we create the sets of equal positions  and different positions , as well as the substrings *s*_1_|*_D _*and . Note that . From these strings, one can infer that  and . To fit *s*_1 _to the solution string *ŝ*, it is necessary to change the positions in *D *without exceeding the limits *d*_1 _and . Thus, for any string *t *of length |*D*| we test whether *t *= *ŝ*|*_D _*is a possible solution. Hence, the width of the search tree is based on the number of strings *t *that fulfill the condition

For these eligible strings *t*, we obtain a new branch of the search tree by creating a new NEIGHBOR STRING instance. The new distance thresholds *e_i _*depend on the distance of *t *to the substrings *s_i_*|*_D_*, so *e_i _*:= *d_i - _d*_H_(*t*, *s_i_*|*_D_*). For *e*_1 _we have the additional constraint . For further information about this approach, see [9].

**The algorithm of Gramm et al**. is a depth-bounded search tree that is initialized with any *s *∈ {*s*_1_,..., *s_k_*}, which is adapted step by step to the solution. The first step is to find a string *s_i _*that differs from the candidate string *s *in more than *d *positions. If no such string exists, then *s *is a valid solution. Otherwise, we change *s *until a center string *ŝ *is found or more than *d *positions in *s *are changed. This results in a maximum tree height of *d*. From the set  we choose *d *+ 1 positions to branch, leading to a total tree size of (*d *+ 1)*^d ^*= *O*(*d^d^*). Since  and , at most *d *elements from  do not converge to a solution. Therefore, choosing *d *+ 1 elements from  produces at least one exact move. For a detailed description of the algorithm, see [12].

**Integrating the set of solved positions ** into the algorithm of Ma and Sun is straightforward, since we can delete all solved positions and decrease *d_i _*by one for every mismatch.

For the algorithm of Gramm *et al*. we cannot have different *d_i_*, hence we have to test whether or not the position is permanent within the search tree. Assume *s *is the candidate string. For any position *p *from  we set *s*[*p*] := *ŝ*[*p*]. During the algorithm, we ensure that none of these positions is changed. Let  be the set of positions that are not permanent. For each string *s_i _*we can estimate the distance threshold *d_i _*between  and  as described for NEIGHBOR STRING instances. Instead of choosing *d *+ 1 positions to branch, we now have to choose only *d_i _*+ 1 positions from . Given that, for all positions in , the candidate string was set to the value of the solution string, there are no positions  with *s_i_*[*p*] = *ŝ*[*p*], and hence . Since  and  at most *d *- *d *+ *d_i _*positions do not a converge to a solution. Therefore, among the *d_i _*+ 1 possible modifications from , there is at least one that brings us *towards *a solution.

**To integrate ****and **, we exclude branches which cannot produce a valid solution. Branches are pruned by simply testing whether the (partial) string candidate of the search tree conflicts with the information. For a position *p *from a particular NEIGHBOR STRING instance we use *m*(*p*) to denote the corresponding position in the original instance.

In the algorithm of Ma and Sun, when creating all strings *t *of length |*D*|, we test for their consistency with the rules from . Assume *t *= *p*_1 _... *p*_l _with *p_i _*∈ *D*, ≤ 1 *i *≤ *l*. For all *p_i _*∈ *D*, 1 ≤ *i *≤ *l *we check whether there is a rule  and test if the remaining positions in *t *are consistent with the partially solved solution string. If that is not the case, the current *t *will not lead to a valid solution. There is even more information in  that we can use. If we find a *t * that is consistent with , we use the solved positions from all sets , with 1 ≤ *i *≤ |*t*|, to reduce the NEIGHBOR STRING instance for the recursion step. For that reason we build an overlay of all  with *p_i _*∈ *D *to get a new set of solved positions. Furthermore, we can check the consistency of *t *with . For all  we test whether *t *has more inconsistent positions than are allowed. Assume *t *= *p*_1 _... *p_l_*. We count all positions *p_n _*with *m*(*p_n_*) ∈ *E_i, j _*and *s_i_*[*m*(*p_n_*)] ≠ *t*[*n*]. If there are more than *x_i,j _*of these positions, the current *t *is not consistent with  and hence cannot produce a valid solution.

In the algorithm of Gramm *et al*., we can restrict the positions we can choose to branch. Assume *s_k _*is the string with *d*_H_(*s, s_k_*) >*d*. We can only branch over a position *p *if we checked the following condition for all  containing *p*: if *s*[*p*] = *s_i_*[*p*] ≠ *s_k_*[*p*] we would have to change *s*[*p*] to *s_k_*[*p*], thus we would set . For that reason we have to check at how many positions from *E_i,j _*the candidate string *s *differs from *s_i_*. If this number is at least *x_i,j_*, we are not allowed to set a further position *p *to  and hence we interdict branching over *p*. Now, let *p *be the current position to branch at, and set *v *:= *s_i_*[*p*]. If  contains *ŝ_p,v_*, we have to adapt the candidate string *s *to *ŝ_p,v _*before calling the recursion. If *s conflicts *with *ŝ_p,v_*, this branch of the search tree does not lead to a valid solution.

### Algorithm MismatchCount

Even after applying our data reduction rules, we have to solve the remaining instance using an algorithm such as those from [9,12]. In this section we present another such procedure, *MismatchCount*, which is effecient in practice, as we will show below. Given binary strings *s*_1_,..., *s_k _*of length *L *and a distance threshold *d*, the MismatchCount algorithm solves the CLOSEST STRING problem as follows: we iterate through all strings *s *with distance at most *d *to a chosen string *s_i _*-- without loss of generality, we may choose that string to be *s*_1 _= 0 ... 0. This leaves us with a search space of size . We present an enumeration scheme for those *s *that allows effecient testing for the center condition on each candidate and that makes it possible to skip large areas of the search space based on information gained while checking those candidates.

**Figure 1:**
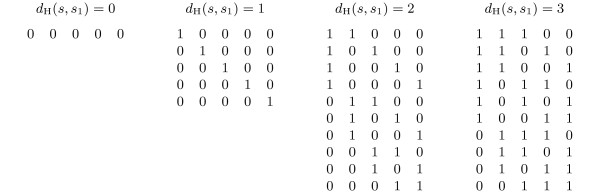
**Enumeration scheme for all strings s**. Enumeration scheme for all strings *s *with Hamming distance at most 3 to a bit string *s*_1 _= 0 ... 0 of length 5.

We enumerate the mismatch positions for *d *mismatches in *s*_1 _(and therefore the center string candidates *s*), which is equivalent to generating all binary numbers of length *m *with *d *bits set to 1, in reverse order (Figure 1). For every *s *we check its Hamming distance to the remaining strings *s*_2_, *s*_3_,..., *s_k_*. Rather than computing these distances anew for each candidate, we update the Hamming distances derived from the previous candidate s'. We do this by increasing or decreasing the distances to reflect the changed positions.

The running time for verifying a center candidate *s *is therefore bounded by *O*(*g *· *k*), where *g *is the number of positions changed from *s*' to *s*.

We can determine the overall number of changes performed during the enumeration of all center candidates as follows: using the enumeration scheme presented, each position *p *in *s *is changed once to '1' and once to '0' for every configuration of *s*[1, *p *- 1] with at most *d *mismatches to *s*_1_[1, *p *- 1]. There are  such configurations for each *d' *= 1, 2,..., *d*. Summing over all possible combinations of *p *and *d'*, the number of bit changes performed can be bounded by *O*(2*^L^*). Since we need to update *k *Hamming distances for each character change in *s*, the overall worst-case running time of the algorithm is bounded by *O*(*k *· 2*^L^*).

However, this worst-case analysis refers to the exploration of all legal mismatch configurations of *s*. As already mentioned above, the enumeration scheme enables us to skip large areas of the search space. Using the maximum Hamming distance *d*_max _= max_*i *= 2,...,*k*_(*d*_H_(*s*, *s*_*i*_)) computed in each iteration, we can derive a lower bound for the number of positions we have to change in *s *in order to fulfill the center condition. Therefore, for each candidate *s *taken into consideration, we compute , where 2 *c*_min _is the minimum number of positions in *s *we have to change when its successor is generated. We can use this condition in two ways. First, we cannot change 2 · *c*_min _positions in *s *by changing the positions of fewer than *c*_min _mismatches. Therefore, if all current candidates *s *with *d*_H_(*s*_1_, *s*) = *d' *are enumerated and we encounter a candidate that reveals a *c*_min _>*d'*, we can then generate candidates with *d*_H_(*s*_1_, *s*) = *c*_min_, without the enumeration of all *s *with *d*_H_(*s*_1_, *s*) ∈ {*d*', *d*' + 1,..., *c*_min _-1}.

**Figure 2:**
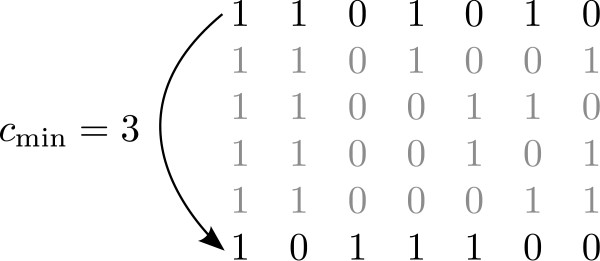
**Skip steps**. Example case where a c_min _value of 3 allows for 4 steps to be skipped.

Furthermore, even if *c*_min _does not exceed d' for a currently observed candidate, we can use that bound to skip the enumeration of certain candidates, i.e. continue with the enumeration scheme where the *c*_min_-th mismatch from the right is moved next (Figure 2). The enumeration steps in between can be omitted because they involve moving fewer than *c*_min _mismatch positions and we know that we have to change at least 2 · *c*_min _positions in *s*.

Applying the data reduction to this algorithm is straightforward. Recall that  is the set of positions that are not permanent. Then, the reduced instance is . When estimating for every candidate *s *its Hamming distance to each remaining string *s_i_*, we have to add the additional amount  to the distances of the reduced strings. This is done only once at the beginning, since we update the Hamming distances during the algorithm.

Within the other algorithms we use the information from  and  to cut off branches of the search tree that cannot contain a valid solution. However, MismatchCount uses an iterative search strategy and positions are not going to be fixed, but can be inverted again. Therefore the use of  and  to fix positions interferes with the use of *c*_min _to skip the enumeration of certain candidates.

## Results and Discussion

### Generating center instances

To evaluate our algorithms, we use instances generated in the context of finding *approximate gene clusters*. The order of genes in genomes can be used to determine the function of unknown genes, as well as the phylogenetic history of the organisms. On this global scale, each gene is represented by one character (or number), and orthologous genes are mapped to the same character. Gene clusters are sets of genes that occur as single contiguous blocks in several genomes. Unfortunately, the requirement of exact occurrences of gene clusters turns out to be too strict for the biological application. This leads to the development of the center gene cluster model [7], which we recapitulate shortly in the following.

Let *S*_1_,..., *S_k _*be the genome strings, where each character represents a gene from the alphabet Σ. Let *S_j_*[*l_j _*... *r_j_*] denote the substring of *S_j _*from position *l_j _*to position *r_j_*. Let  be the *set *of genes in a string *S *∈ Σ*. Finally, let *D *be the symmetric set distance, *D*(*C*, *C' *) = |*C *\*C' *| + |*C' *\*C*|. For some distance threshold *δ*, the *center gene cluster *model asks for all gene clusters *C *⊆ Σ of some minimal size such that, for each *j *∈ {1,..., *k*}, there exist *l_j_*, *r_j _*with

Now, the important point is that the algorithm for center gene cluster detection [7], computes candidates instead of directly finding center gene clusters. These candidates are intervals [*l*_1_, *r*_1_],..., [*l_k_*,..., *r_k_*] such that the sets * might *allow for some center *C *⊆ Σ with *D*(*C*, *C_j_*) ≤ *δ *for all *j *= 1,..., *k*. Our task is to check if the resulting center does indeed meet the distance threshold.

We can transform the approximate occurrences *C_j_*, for *j *= 1,..., *k*, to binary state strings by iterating over all genes that appear in at least one approximate occurrence, using '1' if the approximate occurrence contains the gene, and '0' if it does not. The order of genes is not important in this transformation, but has to be identical for all strings, see also the Preliminaries. Searching for a center gene cluster under the symmetric set distance, is equivalent to searching for a binary string in the transformed instance under the Hamming distance.

The resulting instances are often rather "short", as most approximate gene clusters contain only few genes. To construct longer and, hence, harder instances for our evaluation, we simply concatenate several of these short instances (that are blocks of *k *binary strings) into one long instance, being a single block of *k *binary strings. This allows us to evaluate the performance of the different methods at the borderline between "tractable" and "intractable" instances. At the same time, we argue that the resulting instances are still "biologically valid."

For our evaluation, we use genomes from the NCBI Genome database http://www.ncbi.nlm.nih.gov/sites/entrez?db=genome. Grouping of genes into gene families is done based on the cluster of orthologous groups categorization http://www.ncbi.nlm.nih.gov/COG/. We used two protocols to construct the two data sets, where we believe the second data set to be closer to the biological application that we have in mind.

For the first data set we used five *γ*-proteobacteria (Table 1). Each approximate gene cluster instance consists of five approximate occurrences, one on each genome. An approximate gene cluster instance is converted to five binary strings, as described above. We concatenated instances (each consisting of five strings) until the desired length *L *was reached. Additional strings were constructed in the same fashion, incorporating further cluster occurrences. We created up to 50 instances for each combination of *k *and *L *with *k *= 20, 30, 40, 50 and *L *= 250, 300,..., 500.

**Table 1:** Genomes from the NCBI Genome database for first data set.

Species name	Refseq	Genes	PC
*Buchnera aphidicola *str. APS	NC_002528	607	564
*Escherichia coli *str. K-12 substr. MG1655	NC_000913	4493	4149
*Haemophilus in uenzae *Rd KW20	NC_000907	1789	1657
*Pasteurella multocida *subsp. multocida str. Pm70	NC_002663	2092	2015
*Xylella fastidiosa *9a5c	NC_002488	2838	2766

Five *γ *-proteobacteria from the NCBI Genome database, used for detection of approximate gene clusters to generate biological instances of the center string problem. 'Refseq' is the reference sequence from NCBI Genome database, 'PC' the number of protein-coding genes.

We generated the second data set using 43 genomes (Table 2). To obtain larger instances, we concatenated smaller instances until a pre-defined length *L *was reached. We created 100 instances for each combination of *k *and *L *with *k *= 4, 6, 8, 10 and *L *= 20, 25,..., 40. Note that we do not "concatenate instances vertically", so the resulting instances are probably closer to the "biological truth" than those of the previous protocol.

**Table 2:** Genomes from the NCBI Genome database for second data set.

Species name	Refseq	Genes	PC
*Aquifex aeolicus*	NC_000918	1580	1529
*Clostridium acetobutylicum *ATCC 824	NC_003030	3843	3671
*Corynebacterium glutamicum *ATCC 13032	NC_003450	3073	2993
*Deinococcus radiodurans *R1 chromosome 1,	NC_001263	2687	2629
*Deinococcus radiodurans *R1 chromosome 2	NC_001264	369	268
*Fusobacterium nucleatum*	NC_003454	2125	2063
*Listeria innocua *Clip11262	NC_003212	3065	2968
*Mesorhizobium loti*	NC_002678	6804	674
*Mycoplasma genitalium*	NC_000908	524	475
*Mycoplasma pneumoniae*	NC_000912	733	689
*Mycoplasma pulmonis*	NC_002771	815	782
*Mycobacterium tuberculosis *CDC1551	NC_002755	4293	4189
*Ralstonia solanacearum*, megaplasmid	NC_003296	1684	1676
*Ralstonia solanacearum*	NC_003295	3503	3437
*Rickettsia conorii str*. Malish 7	NC_003103	1414	1374
*Salmonella typhimurium *LT2	NC_003197	4620	4423
*Staphylococcus aureus *subsp. aureus N315	NC_002745	2664	2583
*Synechocystis *sp. PCC 6803	NC_000911	3229	3179
*Thermotoga maritima*	NC_000853	1928	1858
*Ureaplasma urealyticum*	NC_011374	695	646
*Bacillus halodurans *C-125	NC_002570	4170	4065
*Bacillus subtilis*	NC_014479	4170	4062
*Borrelia burgdorferi*	NC_001318	890	851
*Buchnera *sp. APS	NC_002528	607	564
*Campylobacter jejuni*	NC_008787	1707	1653
*Caulobacter crescentus*	NC_002696	3819	3737
*Chlamydia pneumoniae*	NC_000922	1122	1052
*Chlamydia trachomatis*	NC_000117	940	895
*Escherichia coli *O157:H7	NC_002695	5371	5229
*Escherichia coli *str. K-12 substr. MG1655	NC_000913	4493	4149
*Haemophilus influenzae *Rd	NC_000907	1789	1657
*Helicobacter pylori *26695	NC_000915	1627	1573
*Helicobacter pylori *str. J99	NC_000921	1534	1488
*Lactococcus lactis*	NC_002662	2425	2321
*Xylella fastidiosa*	NC_002488	2838	2766
*Neisseria meningitidis *serogroup B str. MC58	NC_003112	2225	2063
*Pasteurella multocida *PM70	NC_002663	2092	2015
*Pseudomonas aeruginosa *PA01	NC_002516	5669	5566
*Rickettsia prowazekii *str. Madrid E	NC_000963	888	835
*Streptococcus pneumoniae*	NC_012467	2254	2073
*Streptococcus pyogenes *str. SF370 serotype M1	NC_002737	1810	1696
*Treponema pallidum*	NC_000919	1095	1036
*Vibrio cholerae *chromosome 1	NC_012668	2897	2768
*Vibrio cholerae *chromosome 2	NC_012667	1013	1004
*Neisseria meningitidis *serogroup A str. Z2491	NC_003116	2065	1909
*Mycobacterium leprae *str. TN	NC_002677	2770	1605

Genomes from the NCBI Genome database used for detection of approximate gene clusters to generate biological instances of the center string problem. 'Refseq' is the reference sequence from NCBI Genome database, 'PC' the number of protein-coding genes.

To compute *d*_opt _we have to increase *d *stepwise, starting from the lower bound for *d*_opt_, given by . We removed all instances that could not be decided for any *d *with *d*_lower _≤ *d *≤ *d*_opt _within a time limit of 10 minutes by any of the algorithms, since we cannot determine the right *d*_opt_. This left us with 664 instances for the first data set and 1957 instances for the second one.

### Removing trivial columns

To avoid taking trivial columns into account, we kept only the dirty columns, representing the "hard part" of the instances. We use *L*' to denote the length of these reduced instances. We stress that in the following, all computations and evaluations are performed on these reduced instances. The amount of reduction shows the difference between the two data sets. While in the first data set we only kept between 35.7% and 56.5% dirty columns, the instances from the second data set are much harder, containing on average between 89.0% and 97.0% dirty columns, depending on the number of strings. The number of dirty columns increases with the number of strings (Table 3).

**Table 3:** Average percentage of dirty columns depending on *k*.

data set	first (5 species)				second (43 species)			
number of sequences *k*	20	30	40	50	4	6	8	10
dirty columns	35.7	43.9	50.4	56.5	89.0	93.8	95.3	97.0

We concentrate on the computation of center strings for *d *= *d*_opt _and *d *= *d*_opt _- 1, since these are the computationally hard instances (Figure 4). For the parameterized algorithms, worst-case running times grow exponentially in *d*, and running times of algorithms are also dominated by these cases in practice.

### Excluding unsolvable instances by preprocessing

Our preprocessing allows us to exclude unsolvable instances more efficiently than the computation of the naïve kernel, when *d *is too small for a center string to exist. This is of particular interest as, here, our algorithms have to scan the complete solution space to ensure that no solution exists. Recall that, during the computation of the naïve kernel, instances with more than *kd *dirty columns or  are rejected, since the instance cannot have a solution for this choice of *d*. The percentage of instances excluded by preprocessing for *d *= *d*_opt _- 1 ranges between 50 and 100 (Table 4). Our improved preprocessing always filters out more instances than does the naïve kernel. For different *k*, we can exclude between 96.2% and 100% of instances, that have not been filtered by the naïve kernel for the first data set and, for the second data set, we can exclude between 87.7% and 94.3% of instances. Recall that the instances we removed (261 for the first data set and 43 for the second one) have not been filtered by preprocessing for their lower bound *d*. Since we cannot determine whether this lower bound is the real *d*_opt _or *d*_opt _- 1, these instances are not taken into account, leading to the high percentages in the first data set.

**Table 4:** Percentage of instances excluded by preprocessing, for *d *= *d*_opt _- 1.

data set	first (5 species)				second (43 species)			
number of sequences *k*	20	30	40	50	4	6	8	10
naïve kernel (%)	81.3	82.2	85.0	86.1	92.9	68.4	56.6	50.0
our preprocessing, from remaining (%)	100	100	96.2	100	94.3	89.6	87.7	90.6
total excluded instances (%)	100	100	99.4	100	99.6	96.7	94.7	95.3

### Solving trivial positions by preprocessing

**Figure 3:**
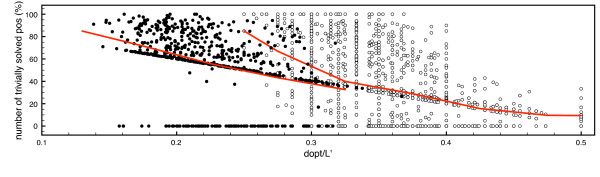
**Percentage of trivially solved positions for *d *= *d*_opt_**. Percentage of trivially solved positions for *d *= *d*_opt _plotted against the *d*_opt _/*L*^' ^ratio of the instances for the first data set (full dots) and the second data set (empty dots). Dots represent individual instances, the solid line is average percentage for intervals of width 0.05.

The second advantage of our method is the computation of positions that can be trivially solved during preprocessing. The percentage of fixed positions is high for the important case *d *= *d*_opt_. In fact, for the first data set an average of 56.2% of the positions were fixed for these instances during preprocessing, and 31.7% for the second data set. Recall that *MismatchCount *and the algorithm of Ma and Sun work on these reduced instances. The number of solved positions depends on the *d*_opt _/*L*' ratio of the instance, since at least *L*' - 2*d*_opt _are fixed if a string pair with distance 2*d*_opt _exists, and decreases with increasing *d*_opt _/*L*' (Figure 3). If we use *d*_opt _/*L*' as a measure for the hardness of the instance, the difference between the two data sets is obvious.

For the first data set we further observe that there is no "twilight zone" of fixed positions. In 80.9% of the instances, more than 40% of positions were fixed; in 15.4%, the data reduction did not fix any positions, and in fewer than 3.8% of the instances, we observed a fixing of up to 40% of positions.

### Running times

We have implemented the algorithms of Gramm *et al. *[12], Ma and Sun [9], and the *MismatchCount *algorithm, referred to as "*Gramm*", "*MaSun*" and "*MismatchCount*", respectively. These algorithms do not include any preprocessing beyond the naïve kernel. Name suffix "*Pre*" indicates that preprocessing and algorithm engineering are enabled. For the *MismatchCount *algorithm, only the information from  is used.

We implemented all algorithms in Java and compiled them with the Sun Java Standard Edition compiler version 1.6. We did all computations on a quad-core 2.2 GHz AMD Opteron processor with 5 GB of main memory under the Solaris 10 operating system. The running times presented are the core running times of the algorithms and do not include I/O or removal of clean columns. We restricted running time to 10 minutes per instance.

**Figure 4:**
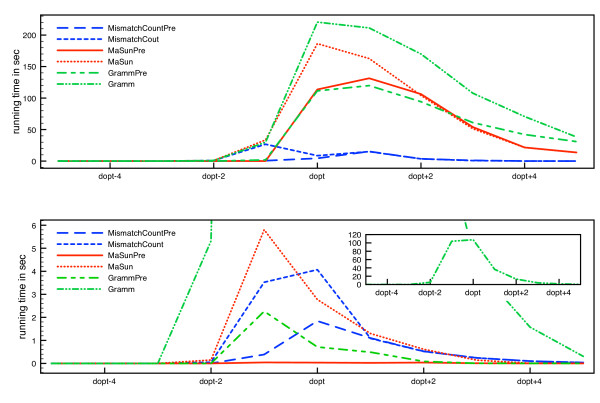
**Average running times of all instances**. Average running times of all instances for first (top) and second (bottom) data set. Running times are depicted in dependency on varying *d *around *d*_opt_. Algorithm *Gramm *is shown separately for the second data set due to the long running times.

We first show that running times of all algorithms are really dominated by the cases *d *= *d*_opt _- 1 and *d *= *d*_opt _(Figure 4). It is clear that it is sufficient to concentrate on the two cases *d *= *d*_opt _- 1 and *d *= *d*_opt_. The short running times for *d*_opt _- 1 for the first data set are again due to the removal of instances for which the lower bound could not be decided. Note that if there is no string pair with distance 2*d*_opt _or 2*d*_opt _- 1, we cannot avoid calling the algorithm with *d *= *d*_opt _- 1 to ensure that *d*_opt _is truly optimal.

To show how running times depend on *d*_opt_, we pooled instances with respect to the optimum center distance d_opt_. For *d *= *d*_opt _- 1 we excluded all instances where *d *<*L*'/*k *after removing clean columns, or  as these obviously have no solution, leaving us with 644 instances for the second data set, while the 108 remaining instances for the first data set are not enough to analyze. Even if instances are not rejected by preprocessing, the algorithms tend to reject instances more quickly if the preprocessing information is used. Different percentages were rejected by the algorithms within different sets of time limits for the second data set (Table 5).

**Table 5:** Percentage of instances excluded by the algorithms within different time limits, for *d *= *d*_opt _- 1.

	*MCPre*	*MC*	*MaSunPre*	*MaSun*	*GrammPre*	*Gramm*
time limit 10 min (%)	100	100	100	100	98.5	9.0
time limit 1 min (%)	98.5	98.5	100	98.5	83.6	4.5
time limit 1 sec (%)	34.3	23.9	89.6	17.9	34.3	1.5

Only the 67 instances of the second data set not rejected by the preprocessing were taken into account. '*MC*' denotes the *MismatchCount *algorithm.

**Figure 5:**
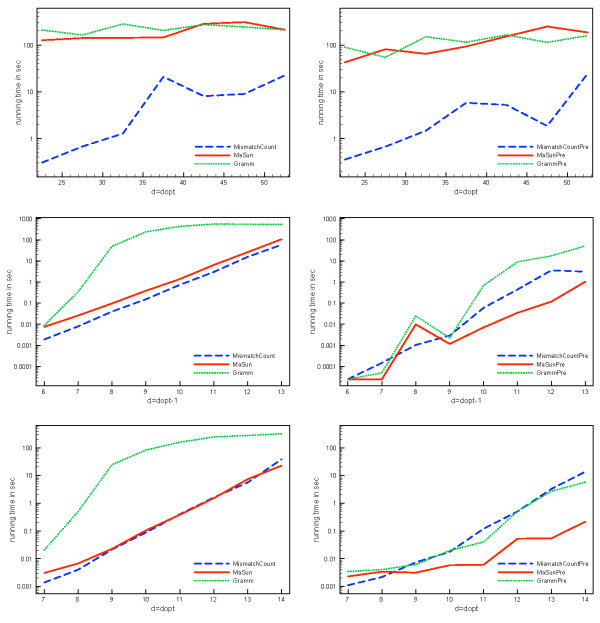
**Average running times for varying *d*_opt_**. Running times for the original (left) and improved implementations (right) for *d *= *d*_opt _for the first data set (top), and *d *= *d*_opt _- 1 (middle) as well as *d *= *d*_opt _(bottom) for the second data set. Note the logarithmic scale for running times.

Using data reduction and information gained during preprocessing reduces the running times of the algorithms for both *d *= *d*_opt _- 1 and *d *= *d*_opt _in all cases (Figure 5). On the first data set, *MismatchCount *using the preprocessing information outperforms the other algorithms, while *MaSunPre *is best on the second data set, especially where *d *= *d*_opt_. The improvement of *MismatchCount *is least significant since the information from  and  cannot be used.

## Conclusions

We have presented improved preprocessing for the CENTER STRING problem. This is based on the observation that, for strings with an optimal center at distance *d*, there are usually many pairs of strings with distance close or equal to 2*d*. Our data reduction allows us to reject more instances that do not have a valid center string, and to draw conclusions about certain positions of a center string. We show how this information can be used in the search tree algorithms of Gramm *et al*., and Ma and Sun. We have also presented the *MismatchCount *algorithm for binary alphabets.

In our experimental evaluation, we showed that, without preprocessing, the *MismatchCount *algorithm has better running times than the other two algorithms. Furthermore, our data reduction is very efficient and algorithms using this information outperform the original ones, with the overall best performance shown by *MismatchCount *on the first data set and the algorithm of Ma and Sun in combination with our preprocessing on the second. Our data reduction is particularly helpful for tackling the case *d *= *d*_opt _- 1, as we can exclude more instances.

For the Levenshtein distance and weighted edit distances, the CENTER STRING problem problem is W[1]-hard regarding the number of input strings. To the best of our knowledge, it is an open problem if these problems are W[1]-hard regarding the distance parameter, too. In this case, our parameterized methods would be not applicable for these distances.

## Authors' contributions

FH and SB jointly developed the data reduction methods. KJ, LK and JS jointly developed the MismatchCount algorithm. FH carried out the computational studies and drafted the manuscript. All authors read and approved the final manuscript.

## References

[B1] Lanctot JK Li M Ma B Wang S Zhang L Distinguishing string selection problems Information and Computation 2003 185 41 55 http://www.sciencedirect.com/science/article/B6WGK-48D37KJ-3/2/219c09ad466c21ba5d31e8f20793ce48 10.1016/S0890-5401(03)00057-9

[B2] Wang L Zhu B Effective Algorithms for the Closest String and Distinguishing String Selection Problems Proc. of Frontiers in Algorithmics Workshop (FAW 2009), Volume 5598 of Lect. Notes Comput. Sc., Springer 2009 261 270 http://www.springerlink.com/content/k2086p4131001276/

[B3] Wang Y Chen W Li X Cheng B Degenerated primer design to amplify the heavy chain variable region from immunoglobulin cDNA BMC Bioinformatics 2006 7 Suppl 4 S9 10.1186/1471-2105-7-S4-S9 17217527 PMC1780117

[B4] Davila J Balla S Rajasekaran S Fast and Practical Algorithms for Planted (*l, d*) Motif Search IEEE/ACM Trans. Comput. Biol. Bioinformatics 2007 4 4 544 552 10.1109/TCBB.2007.70241 17975266

[B5] Yanai I DeLisi C The society of genes: networks of functional links between genes from comparative genomics Genome Biol 2002 3 11 research0064 12429063 10.1186/gb-2002-3-11-research0064 PMC133448

[B6] Rahmann S Klau GW Mandoiu I, Zelikovsky A Integer linear programming techniques for discovering approximate gene clusters Bioinformatics Algorithms: Techniques and Applications, Wiley Series on Bioinformatics: Computational Techniques and Engineering 2008 Wiley 203 222

[B7] Böcker S Jahn K Mixtacki J Stoye J Computation of median gene clusters J. Comput. Biol 2009 16 8 1085 1099 10.1089/cmb.2009.0098 19689215

[B8] Frances M Litman A On covering problems of codes Theory Comput. Systems 1997 30 2 113 119

[B9] Ma B Sun X More Effective Algorithms for Closest String and Substring Problems SIAM J. Comput 2009 39 4 1432 1443 http://link.aip.org/link/?SMJ/39/1432/1

[B10] Liu X He H Sykora O Parallel Genetic Algorithm and Parallel Simulated Annealing Algorithm for the Closest String Problem Proc. of Advanced Data Mining and Applications Conference (ADMA 2005), Volume 3584 of Lect. Notes Comput. Sc., Springer 2005 591 597 http://www.springerlink.com/content/4quu3p835k4d2txp/

[B11] Faro S Pappalardo E Ant-CSP: An Ant Colony Optimization Algorithm for the Closest String Problem Proc. of Current Trends in Theory and Practice of Computer Science (SOFSEM 2010), Volume 5901 of Lect. Notes Comput. Sc., Springer 2010 370 381

[B12] Gramm J Niedermeier R Rossmanith P Fixed-parameter algorithms for Closest String and related problems Algorithmica 2003 37 25 42 10.1007/s00453-003-1028-3

[B13] Chen ZZ Ma B Wang L A Three-String Approach to the Closest String Problem Proc. of Computing and Combinatorics Conference (COCOON 2010), Volume 6196 of Lect. Notes Comput. Sc., Springer 2010 449 458

[B14] Meneses C Lu Z Oliveira C Pardalos P Optimal solutions for the closest-string problem via integer programming INFORMS J. Computing 2004 16 4 419 429 10.1287/ijoc.1040.0090

[B15] Kelsey T Kotthoff L The Exact Closest String Problem as a Constraint Satisfaction Problem Computing Research Repository 2010 **abs/1005.0089**

[B16] Nicolas F Rivals E Hardness results for the center and median string problems under the weighted and unweighted edit distances Journal of Discrete Algorithms 2005 3 390 415 10.1016/j.jda.2004.08.015

